# Cognitive behavioral therapy for reducing fear of cancer recurrence (FCR) among breast cancer survivors: a systematic review of the literature

**DOI:** 10.1186/s12885-021-08909-y

**Published:** 2022-02-28

**Authors:** So-Young Park, Jung-Won Lim

**Affiliations:** 1 Ewha Institute for Age Integration Research, Ewha Womans University, 52 Ewhayeodae-gil, Sedaemun-gu, Seoul, 03760 Republic of Korea; 2 College of Social Welfare, Kangnam University, 40 Kangnam-Ro, Giheung-Gu, Yongin-Si, Gyeonggi-Do 16979 Republic of Korea

**Keywords:** Breast cancer, Cognitive behavioral therapy, Fear of recurrence, Randomized controlled trials

## Abstract

**Background:**

Fear of cancer recurrence (FCR) has been addressed as a cause of emotional distress among breast cancer survivors (BCSs). This study aimed to systematically review the evidence on randomized controlled trials (RCTs) of cognitive behavioral therapy (CBT) designed to reduce FCR among BCSs.

**Methods:**

A systematic review of published original research articles meeting the inclusion criteria was conducted. Five electronic databases, including the Cochrane Library, CINAHL, PubMed, PsycINFO, and Web of Science, were independently searched to identify relevant articles. The Consolidated Standards of Reporting Trials (CONSORT) 2010 checklist was used to evaluate the quality of the eligible studies.

**Results:**

Through a database search and a manual review process, seventeen quantitative studies with an RCT study design were included in the current systematic review. The interventions varied greatly in length and intensity, but the study designs and methodologies were similar. RCTs with face-to-face interventions of at least 1 month seemed to be more effective in reducing FCR outcomes and complying with than the CONSORT 2010 criteria than those with a brief online or telephone format of interventions; nevertheless, most RCT interventions appeared to be effective.

**Conclusions:**

These findings highlight the importance of conducting well-designed CBT interventions to reduce FCR in BCSs with diverse populations at multiple sites, thereby improving the quality of research in this area.

**Supplementary Information:**

The online version contains supplementary material available at 10.1186/s12885-021-08909-y.

## Background

Breast cancer is the most frequent cancer among women, with an estimated 276,480 new cases of invasive breast cancer diagnosed among women in the United States (U.S.) in 2020 [1]. With earlier detection and more effective treatments, the death rate decreased by 1.3% per year from 2013 to 2017 [2]. As a result, the number of breast cancer survivors (BCSs) continued to increase, with BCSs constituting the largest population of cancer survivors, at an estimated 2.6 million women in the U.S. [1]. Although BCSs live longer, they are at risk for physical, psychological, and social symptoms associated with illness and its treatment [3, 4]. Several studies have reported that psychological and emotional distress is likely to increase the experience of pain in BCSs and reduce social performance and overall quality of life [5]. Thus, long-term and late effects during the cancer survivorship phase require posttreatment survivorship care.

Of the diverse forms of psychological distress that BCSs experience, fear of cancer recurrence (FCR) is known to be one of the most prevalent, persistent, and disruptive problems [6–8]. FCR is generally defined as “fear, worry, or concern about cancer returning or progressing” [8]. Approximately 50% of BCSs report moderate-to-high FCR levels [9]. A few studies have also reported that up to 70% of BCSs experience FCR, which is associated with long-term functional impairments [10, 11].

FCR can negatively influence screening, health behaviors, mood, coping behaviors, and quality of life [10, 12]. BCSs also tend to report an unmet need for help with FCR at treatment completion, suggesting that the management of FCR may be their greatest unmet need [8, 13]. More specifically, previous studies have showed that BCSs with FCR use maladaptive coping strategies, such as excessive reassurance seeking, anxious avoidance, intrusive thoughts, denial, or self-blame [14–16]. For example, intrusive thoughts about the cancer and treatment occurred even years after the completion of treatment [17]. Although BCSs are expected to live longer after cancer treatment, if their psychological distress is left untreated, debilitating fears may continually influence their remaining lives, thereby reducing their quality of life [8, 18].

Since FCR is significant as a cause of emotional distress among BCSs, FCR should be addressed as a logical, clinically relevant target for intervention [19, 20]. Given that FCR is associated with coping behaviors and unmet needs, cognitive behavioral therapy (CBT) may be a common psychotherapeutic intervention for BCSs with FCR [21]. Indeed, CBT has proven to be more effective than usual care in reducing FCR, with reported effect sizes of − 0.20 to − 0.73 [22–24]. There is a growing body of research on interventions based on CBT for FCR. Although the theoretical foundations, formats and delivery methods of these interventions differ, the available interventions are based on CBT [25]. For example, the effectiveness of CBT, including problem-solving therapy and behavioral activation, in reducing FCR has been shown among BCSs, indicating that problem-solving therapy contributes to survivors’ better coping with situations that commonly trigger FCR [21]. CBT interventions, including mindfulness stress reduction, acceptance and mindfulness, and compassion-based interventions were also known to improve FCR for BCSs [24, 26, 27]. That is, acceptance and commitment therapy, which emphasizes acceptance while living mindfully according to one’s values, was effective in facilitating the adaptive management of FCR for BCSs [28]. Mindfulness-based interventions tend to emphasize awareness to induce physiological relaxation and help individuals emotionally disconnect from depressing thought patterns [29, 30]. Recently, several studies found that mindfulness-based interventions were effective as coping strategies that diminish anxiety, stress, and general mood and enhance quality of life for BCSs [31, 32]. The evidence on compassion-based interventions designed to generate cognitive compassionate habits also suggests that they can provide useful skills to prevent FCR for BCSs [33].

Tauber and colleagues [34] evaluated the effects of psychological intervention on FCR in a systematic review and meta-analysis, and indicated that larger postintervention effects were found for interventions that were focused on the processes, rather than the content of cognition. Given that Tauber and colleagues [34] included both controlled and open trials among patients with and survivors of cancer, systemic reviews of more rigorous interventions focusing on randomized controlled trials (RCTs) are required to provide a comprehensive overview of current knowledge on CBTs designed to reduce FCR among BCSs. Additionally, earlier studies on CBTs have not fully considered methodological approaches based on the Consolidated Standards of Reporting Trials (CONSORT) 2010 statement [35]. Systemic reviews on CBTs using rigorous methodologies will be helpful to fill gaps in the literature by examining the reported effects of CBTs on FCR for BCSs.

The purpose of this study is to conduct a systematic review of RCTs with CBT interventions for reducing FCR among BCSs who have completed active treatment. More specifically, this systematic review study evaluates RCT interventions with regard to the content and methodological aspects of the interventions, the FCR outcomes, and the quality of the studies.

## Methods

### Search strategy and sources

The Preferred Reporting Items for Systematic Reviews and Meta-Analyses (PRISMA) statement [36] was used as a basis for screening and selecting studies. The Cochrane Library, CINAHL, PubMed, PsycINFO, and Web of Science databases were systematically searched between July 13, 2020, and July 15, 2020, to identify relevant studies. The key search terms were (breast) AND (cancer OR carcinoma OR neoplasm) AND (fear* OR concern OR worr* OR anxiet*) AND (recur* OR relapse OR progress*) AND (cognitive behav* therap*). One of the authors (S.P.) performed all searches. In addition to the database search, the bibliographies of all included articles were manually screened to identify other relevant articles.

### Selection strategy

To select eligible studies, the following PICOTS-SD criteria were applied: (1) Participants (P): female breast cancer survivors who had completed active treatment (e.g., surgery, chemotherapy, etc.); (2) intervention (I): CBT interventions; (3) comparison (C): usual care, other psychological intervention, or no intervention; (4) outcome (O): quantitative FCR outcomes using FCR-related measures; (5) time (T): pretest, posttest, and follow-up; (6) setting (S): hospitals or community-based organizations; and (7) study design (SD): RCTs. Furthermore, the studies had to be written in English and published from January 2010 to July 2020. We excluded review articles, books and book chapters, qualitative studies, commentaries, editorials, poster abstracts, case reports, articles on childhood survivorship populations or without control groups, and original studies without full texts.

### Data extraction

First, the titles and abstracts of the potential eligible records were reviewed. Second, duplicates and unsuitable articles were removed from the records. Then, the articles that fulfilled the inclusion criteria were subsequently obtained in full text and examined by the authors (S.P. and J.L.). Any discrepancies regarding a study’s inclusion or exclusion were discussed as a group and were resolved by consensus. For each study, two authors (S.P. and J.L.) extracted the first author’s name, publication year, country of study, sociodemographic and cancer-related characteristics, sample size, study design, description of the CBT interventions, FCR measures, and summary of the primary outcome findings.

### Quality assessment

The CONSORT 2010 statement [35] was utilized to assess the methodological quality of all included studies. The CONSORT 2010 statement is a 37-item checklist that includes all aspects of reporting, such as the title and abstract, introduction, methods, the results, discussion, and other information. In the present review, four of 37 items (e.g., any changes to trial outcomes after the trial commenced, with reasons [6b]; if relevant, description of the similarity of interventions [11b]; why the trial ended or was stopped [14b]); and for binary outcomes, presentation of both absolute and relative effect sizes is recommended [17b]) were not used for quality assessment because they were not applicable to the included studies. The authors reviewed the selected articles according to the CONSORT 2010 checklist and rated them as “yes” or “no.” If the articles described each of the 33 checklist items, they were categorized as “yes.” In contrast, if the articles did not report adequate information or lack of information on these items, they were evaluated as “no.” Then, the number and proportion of the included studies reporting each applicable item on the checklist were calculated. Any disagreements were discussed by the authors (S.P. and J.L.) until consensus was reached.

## Results

### Study selection

**Fig. 1:**
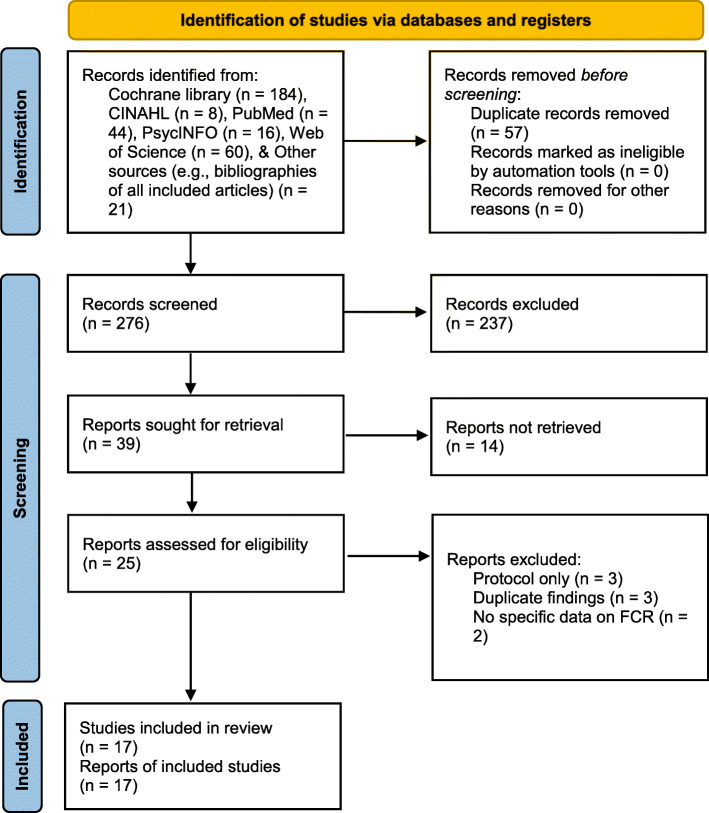
PRISMA Flow Diagram of the Literature Search

A total of 333 studies were extracted from the online databases and other sources (e.g., bibliographies of all included studies). After the removal of duplicates and ineligible records, 276 records were screened. Based on the evaluation of the titles and abstracts, 237 articles were excluded because they were not relevant, with common reasons for exclusion (e.g., no breast cancer survivors, no RCT study design, no CBT intervention, no data on FCR, and abstract only). Then, 25 full-text articles were assessed for secondary screening, of which eight studies were excluded for the reasons described in Fig. 1. After a review and discussion among authors, 17 articles were finally selected for the systematic review (see Fig. 1 for PRISMA flow diagram of the literature review).

### General characteristics of the interventions

A summary of the general characteristics of the study participants, including the sample size, age, sex, ethnicity, cancer type, cancer stage, and time since diagnosis, is listed in Table 1. Of the included studies, most studies were conducted in the U.S. (*n* = 8), the Netherlands (*n* = 2), or Germany (n = 2), and other countries represented were Spain, Belgium, Canada, Australia, and Japan. The total sample size across the studies was 2288 participants, with the sample sizes of each study ranging from 24 to 322. The participants in the sample were predominately non-Hispanic White females, with an average age of 53.1 years. Most studies focused on only breast cancer survivors (*n* = 12), while some studies included mixed cancer populations (*n* = 5). Most of the studies recruited cancer survivors diagnosed at stages 0 to 3 or stages 1 to 3, but two studies included people with stage 4 cancer. The means of time since diagnosis for the experimental and control groups were 4.48 and 4.49 years, respectively, which indicated that the study participants had completed their treatment rather recently.

**Table 1:** General characteristics of the reviewed articles

Author, Country	Sample size (Total Sample)	Age at Survey (Mean, SD)	Sex	Ethnicity	Cancer Type	Cancer Stage	Time Since Diagnosis in Years (Mean, SD)
Herschbach et al. [23], Germany	174	Total: 53.7(10.2), CBT: 53.7(9.6), SET: 53.8(10.6), Control: 53.7(10.3)	85.7% female	German	Breast, colorectal, bladder/prostate, gynecological, etc.	No information	No information
van de Wal et al. [24], The Netherlands	88	Total: 58.9, CBT: 58.0(11.3), CAU: 59.7(10.0)	53% female	Dutch	Breast, prostate, and colorectal cancer	No information	CBT: 2.4(1.5), CAU: 2.8(1.3)
Butow et al. [26], Australia	222	Total: 52.8(10.1), ConquerFear 53.3(10.5), Control 52.3(9.6)	95% female	Australian	Mixed (breast, colorectal, or melanoma)	0-3	ConquerFear (median): 2.26, control(median):2.43
Lengacher et al. [27], USA	322	Total: 56.6(9.7), MBSR: 57.6(9.2), Control: 56.5(10.2)	100% female	69.4% non-Hispanic Whites	Breast	0-3	No information
Bower et al. [37], USA	71	Total: 46.9 MAPS: 46.1, Control: 47.7	100% female	76.1% Non-Hispanic Whites	Breast	0-3	MAPS: 4.0(2.4), Control: 4.1(2.3)
Johns et al. [38], USA	91	Total: 58.7(10.7), ACT: 59.8(11.1), SE: 57.5(10.5), EUC: 58.7(10.5)	100% female	83.52% non-Hispanic Whites	Breast	1-3	No information
Dodds et al. [39], USA	28	Total: 55.3, CBCT: 54.7(12.1), Control: 55.8(9.7)	100% female	Non-Hispanic Whites	Breast	1-4	CBCT: 4.8(3.2), Control: 5.8(6.0)
Gonzalez-Hernandez et al. [40], Spain	56	Total: 52.1 (7.0), CBCT: 51.6(6.9), TAU: 52.6(7.2)	100% female	Spanish	Breast	1-3	CBCT: 11.32(1.44), Control: 10.46(2.90)
Lengacher et al. [41], USA	82	Total: 57.2(9.2)	100% female	71.9% non-Hispanic Whites	Breast	0-3	No information
Park et al. [42], Japan	74	Total: 53.7, MBCT: 53.2(8.4), Control: 54.2(9.3)	100% female	Japanese	Breast	0-3	MBCT: 3.27(3.10), Control: 3.43(5.41)
Lichtenthal et al. [43], USA	97	Total: 54.9, AIM-FBCR: 55.8(7.4), Control: 53.9(10.3)	100% female	AIM-FBCR: 73.0% non-Hispanic Whites, Control: 75% non-Hispanic Whites	Breast	0-3	No information
Tomei et al. [44], Canada	24	Total: 55.0 (10.8), CEP: 53.9(13.5), Control: 56.0(8.3)	100% female	95.8% non-Hispanic Whites	Breast, gynecological and ocular melanoma	1-3	Range: 0-6, CEP: 1.91(1.30), Control: 1.23(1.23)
van Helmondt et al. [45], The Netherlands	262	Total: 55.8(9.9), CBT: 55.3(10.1), CAU: 56.2(9.8)	100% female	Dutch	Breast	No information	CBT: 2.5(1.1), Control: 2.6(1.2)
Heinrichs et al. [46], Germany	180	Total: 52.5, Patient: 52.2(11.3), Partner: 52.7(11.4)	Patient group: 100% female	German	Breast or gynecological cancer	0-3	No information
Shields et al. [47], USA	45	Total: 44.1, CBT: 44.2(5.0), Control: 44.0(4.1)	100% female	97.5% non-Hispanic Whites	Breast	1-3	CBT: 5.6(1.7), Control: 5.5(1.6)
Germino et al. [48], USA	313	Total: 44	100% female	117 African American, 196 non-Hispanic Whites	Breast	1-4	No information
Merckaert et al. [49], Belgium	159	Total: 50.6 (10.1)	100% female	Belgian	Breast	1-3	No information

**Table 2:** Content and methodological strategies of the interventions

Author, Country	CBT Intervention Type	Intervention Format	Delivery Mode	Number of Sessions	Session Length	Duration (Weeks)	Interventionist	Study Design	Comparable Group(s)	Randomization (Selection Bias)	External Validity
Herschbach et al. [23], Germany	CBT	Group	Face-to-face	4	1.5 hours	4	Psychotherapists	A two-site, longitudinal controlled design	1) Supportive-experiential group therapy, 2) Control group	Random assignment to one of two interventions, but not for the control group	Two rehabilitation clinics in Southern Germany
van de Wal et al. [24], The Netherlands	Blended CBT	Individual	Face-to-face + Online	8 (5individual+ 3 online)	1 hour + 15 minutes	12	Two psychologists	A parallel-group, prospective randomized controlled trial	Care as usual	Random assignment	Five hospitals in the Netherlands
Butow et al. [26], Australia	Meta-cognitive therapy, Self-Regulatory Executive Function (S-REF) model, and Acceptance and commitment therapy (ACT)	Individual	Face-to-face	5	1 to 1.5 hours	10	Psychologists or psychiatrists	A randomized controlled trial	A nonspecific attention control (Taking-it-Easy: a relaxation training program)	Random assignment	Multiple centers in different areas in Australia
Lengacher et al. [27], USA	MBSR	Group	Face-to-face	6	2 hours	6	Psychologist	A randomized controlled trial	Waitlisted control	Random assignment	Mostly non-Hispanic Whites
Bower et al. [37], USA	Mindful awareness practices (MAPS)	Group	Face-to-face	6	2 hours	6	No information	A single-center, two-armed RCT	Waitlisted control	Random assignment	Mostly non-Hispanic Whites
Johns et al. [38], USA	ACT	Group	Face-to-face	6	2 hours	6	Doctoral-level therapist	A randomized controlled pilot trial	1) Survivor education, 2) Enhanced usual care	Random assignment	Mostly non-Hispanic Whites
Dodds et al. [39], USA	Cognitively based compassion training (CBCT)	Group	Face-to-face	8	2 hours	8	Social work researcher	A randomized wait list-controlled trial design	Waitlisted control	Random assignment	Mostly non-Hispanic Whites
Gonzalez-Hernandez et al. [40], Spain	CBCT	Group	Face-to-face	8	2 hours	8	Psychologist	A randomized controlled trial	Treatment-as-usual control	Random assignment	Conduced in Spain
Lengacher et al. [41], USA	Mindfulness-based stress reduction (MBSR(BC))	Group	Face-to-face	6	2 hours	6	Psychologist	A randomized controlled trial	Waitlisted control	Random assignment	Mostly non-Hispanic Whites
Park et al. [42], Japan	Mindfulness-based cognitive therapy (MBCT)	Group	Face-to-face	8	2 hours	8	Psychologists, psychiatrists, and nurses	A randomized controlled trial	Waitlisted control	Random assignment	Conducted in Japan
Lichtenthal et al. [43], USA	Attention and Interpretation Modification for Fear of Breast Cancer Recurrence	Group	Face-to-face	8	30 minutes	4	No information	A parallel-group randomized trial	Control condition	Random assignment	Mostly non-Hispanic Whites
Tomei et al. [44], Canada	Cognitive-existential psychotherapy (CEP)	Individual	Face-to-face	6	2 hours	6	Psychology doctoral students	A pilot randomized controlled trial	Waitlisted control	Random assignment	Mostly non-Hispanic Whites
van Helmondt et al. [45], The Netherlands	CBT-based online self-help training	Individual	Online	6	no information	12	No information	A randomized controlled trial	Care as usual (psychological or other support)	Random assignment	Eight hospitals in the Netherlands
Heinrichs et al. [46], Germany	Couples-based skills intervention (side by side)	Couple	Face-to-face	4	2 hours	8	Psychologists	A two-site, balanced randomized, controlled, parallel-group design	Control program	Random assignment	Three regional hospitals in Germany
Shields et al. [47], USA	Coaching intervention (prompt plus telephone counseling)	Individual	Telephone	1	20 to 45 minutes	1	Nurse	A randomized pilot trial	Usual care	Random assignment	Small and homogeneous sample
Germino et al. [48], USA	Cognitive and behavioral strategies	Individual	Telephone	4	20 minutes	4~6 months	Nurses	A 2 x 2 randomized block, repeated-measures design	Attention control	Random assignment	non-Hispanic Whites and African Americans
Merckaert et al. [49], Belgium	Cognitive behavioral techniques and hypnosis	Group	Face-to-face	15	2 hours	15	Psychologists	A multicenter randomized controlled trial	Enhanced standard care	Random assignment	Multiple centers in Belgium

### Content and methodological strategies of the interventions

First, the included interventions utilized a wide range of CBT techniques with some variations, such as mindfulness awareness practices (MAPS) [37], acceptance and commitment therapy (ACT) [26, 38], cognitively based compassion training (CBCT) [39, 40], mindfulness-based stress reduction (MBSR) [27, 41], mindfulness-based cognitive therapy (MBCT) [42], attention and interpretation modification [43], cognitive-existential psychotherapy [44], blended CBT [24], and CBT-based online self-help training [45].

More than half of the studies used group-based interventions, while six studies adopted an individual format. One of the group intervention studies targeted breast or gynecological cancer survivors and their partners [46]. Most interventions were delivered face-to-face, and one study combined in-person delivery with an online method [24]. A couple of studies used either telephone communication [47, 48] or online communication [45]. The frequency and duration of the interventions varied greatly, from a single 20- to 45-min session [47] to 15 weekly 2-h sessions [49]. The most common intervention duration was six or 8 weeks (*n* = 9). The CBT interventionists included heath care professionals such as psychotherapists, psychologists, or nurses. The threat of selection bias was low because all the studies utilized a pretest-posttest control group design with random assignment. Most studies included an active comparison group and/or a control group with usual care during the intervention period. In particular, Herschbach et al. [23] compared a CBT group with a comparison group (supportive-experiential group) and a control group, and Butow et al. [26] compared a CBT group with a comparison group that received a relaxation training program. Johns et al. [38] included both a comparison group (e.g., survivorship education) and a control group and compared these groups with a CBT group. Six studies [27, 37, 39, 41, 42, 44] compared a CBT group with a waitlisted control group. Last, there were limitations regarding a lack of external validity (e.g., small and highly homogeneous samples, the same settings and regions in the U.S. or European countries) in most studies. However, Germino et al.’s [48] study targeted both non-Hispanic Whites and African Americans, and Butow et al. [26] recruited samples from 17 oncology centers in Australia.

### FCR instruments

Five different instruments were used to measure FCR in the selected studies: the Cancer Worry Scale (CWS) [50], the Concerns about Recurrence Scale (CARS) [51], the Fear of Cancer Recurrence Inventory (FCRI) [52], the short form of the Fear of Progression Questionnaire (FoP-Q-SF) [53], and the Quality of Life in Adult Cancer Survivors (QLACS)-FCR subscale [54]. Among these instruments, the FCRI (42 items) and the CARS (30 items) were the most frequently used in the included studies. Eight studies [24, 26, 38–40, 44, 45, 49] used either the FCRI total scale or some of the seven FCRI subscales (e.g., triggers [8 items], severity [9 items], psychological distress [4 items], coping strategies [9 items], functioning impairments [6 items], insight [3 items], and reassurance [3 items]). Six studies [27, 41–43, 47, 48] used the 30-item CARS, which is composed of two measures: overall fear and a range of problems. Van de Wal et al. [24] used both the 8-item CWS and FCRI, and other studies measured FCR by using either the 12-item FoP-Q-SF [23, 46] or the 4-item QLACS-FCR subscale [36]. Table 3 provides a summary of instruments used to assess FCR.

**Table 3:** Instruments used to assess fear of recurrence

Name of FCR Instrument	Composite Measure	Number of Items	Response Format	Studies Using Each Measure
Cancer Worry Scale (CWS) [50]		Total: 8	1~4	van de Wal et al. [24]
Concerns about Recurrence Scale (CARS) [51]	1) Overall fear (or concerns)	4	1~6	Germino et al. [48], Lengacher et al. [41], Lengacher et al. [27], Lichtenthal et al. [43], Park et al. [42]
	2) Problems (death, health, role, womanhood, and parenting)	26	0~4	Lengacher et al. [41], Lengacher et al. [27], Lichtenthal et al. [43], Shields et al. [47]
		Total: 30		
Fear of Cancer Recurrence Inventory (FCRI) [52]	1) Triggers	8	0~4	Dodds et al. [39], Gonzalez-Hernandez et al. [40], Johns et al. [38], Merckaert et al. [49]
	2) Severity	9	0~4	Dodds et al. [39], Johns et al. [38] Merckaert et al. [49], van Helmondt et al. [45]
	3) Psychological distress	4	0~4	Dodds et al. [39], Gonzalez-Hernandez et al. [40], Johns et al. [38], Merckaert et al. [49]
	4) Coping strategies	9	0~4	Gonzalez-Hernandez et al. [40], Johns et al. [38], Merckaert et al. [48], van Helmondt et al. [45]
	5) Functioning impairments	6	0~4	Dodds et al. [39], Johns et al. [38], Merckaert et al. [49], van Helmondt et al. [45]
	6) Insight	3	0~4	Dodds et al. [39], Gonzalez-Hernandez et al. [40], Johns et al. [38], Merckaert et al. [49]
	7) Reassurance	3		Johns et al. [38], Merckaert et al. [49]
		Total: 42	0~4	Butow et al. [26], Tomei et al. [44], van de Wal et al. [24]
Short form of the Fear of Progression Questionnaire (FoP-Q-SF) [53]		12	1~5	Heinrichs et al. [45], Herschbach et al. [23]
Quality of Life in Adult Cancer Survivors (QLACS) [54]	Fear of Cancer Recurrence subscale	4	1~7	Bower et al. [37]

### FCR-related outcomes

The FCR-related outcomes of all studies are listed in Table 4. FCR was included as an outcome variable in most studies except Lengacher et al.’s study [41], which treated FCR as a mediator. Three studies [24, 41, 49] assessed FCR only at baseline or pretest (T0) and posttest (T1), and other studies assessed FCR scores at baseline or pretest, posttest, and one or two follow-up periods. Most studies showed significant reductions in the FCR scores of the intervention groups at the postintervention and follow-up time points, but some results were not statistically significant. In particular, three studies [45, 47, 48] reported no significant between-group differences in FCR across time. The common aspects of these studies were a telephone or online format and brief sessions of less than 1 h. Seven studies [23, 26, 27, 41, 42, 44, 46] reported both significant main effects in the intervention groups and significant group-by-time interaction effects on all FCR scores over time. In general, these interventions were in four to eight 60- to 120-min, face-to-face group sessions over at least 4 weeks and the interventionists were trained health care professionals such as psychotherapists and psychiatrists. In addition, seven studies using either the FCRI or the CARS subscales [24, 38–40, 43, 47, 49] showed partially significant improvements in a couple of subscales among the intervention groups. Interestingly, two studies [23, 38] included three arm RCTs to compare CBT with a comparison group (e.g., supportive-experiential group therapy, survivor education) and a control group and they found that supportive-experiential group therapy was comparable to the CBT intervention while survivor education demonstrated minimal changes in reducing FCR over time.

**Table 4:** FCR-related outcomes

Author, Country	FCR Instrument	FCR Variable Type	Assessment Time	Major Findings (Focused on FCR)
Herschbach et al. [23], Germany	FoP-Q-SF	Outcome	Pretest (T0), posttest (T1), 3-month f/u (T2), 12-month f/u (T3)	There was a significant main effect of time and a significant interaction of group x time. FoP decreased significantly over time in both intervention groups but not in the control group.
van de Wal et al. [24], The Netherlands	CWS	Outcome	Baseline (T0), posttest (T1)	The patients in the bCBT group reported significantly lower CWS and FCRI scores (total score, scores for severity/triggers/distress/functioning impairments) than those in the CAU group.
	FCRI: severity, psychological distress, triggers, coping strategies, functioning impairments, insight, & reassurance	Outcome	Baseline (T0), Posttest (T1)	
Butow et al. [26], Australia	FCRI: total	Outcome	Baseline (T0), posttest (T1), 3-month f/u (T2), 9-month f/u (T3)	The ConquerFear participants showed greater improvements in FCRI scores than the control participants.
Lengacher et al. [27], USA	CARS: overall fear, problems	Outcome	Baseline (T0), posttest (T1), 3-month f/u (T2)	MBSR(BC) showed significant improvements in FCRs (overall and problems) than UC group at T1 and T2 periods.
Bower et al. [37], USA	QLACS	Outcome	Baseline (T0), posttest (T1), 3-month f/u (T2)	There was no significant group x time interaction effect on FCR at post intervention but there was a significant group difference (group x time interaction) in FCR at the 3-month follow-up.
Johns et al. [38], USA	FCRI: severity, triggers, distress, functioning impairments, insight, reassurance seeking, and coping strategies	Outcome	Baseline (T0), posttest (T1), 1-month f/u (T2), 6-month f/u (T3)	ACT was associated with significant within-group improvements in FCR severity and in the scores for all secondary FCRI subscales except for reassurance seeking and coping across time; between-group differences favored ACT over survivorship education and enhanced usual care, most obviously at T3.
Dodds et al. [39], USA	FCRI: severity, triggers, psychological distress, functioning impairments, and insight domains	Outcome	Baseline (T0), posttest (T1), 1-month f/u (T2)	Compared to the control condition, CBCT was a feasible intervention and was highly satisfactory to BC survivors. Functioning impairments associated with FCR showed a significant changes between pre- and post intervention for the CBCT group.
Gonzalez-Hernandez et al. [40], Spain	FCRI: triggers, psychological stress, coping strategies, and insight	Outcome	Pretest (T0), posttest (T1), f/u (T2)	Psychological stress showed a significant time x group interaction, but there were no significant interaction effects for other factors. Within-group comparisons showed significant pre-to-post and pre-to-follow-up changes in psychological stress for the CBCT group but no significant changes in the TAU group.
Lengacher et al. [41], USA	CARS: overall fear, problems	Mediator	Pretest (T0), posttest (T1)	MBSR(BC) resulted in significant reductions in FCR and improved physical functioning which, in turn, mediated significant reductions in perceived stress and anxiety.
Park et al. [42], Japan	CARS: overall	Outcome	Baseline (T0), posttest (T1), 3-month f/u (T2)	Compared with the control group, the MBCT group showed significant reductions in FCR over time.
Lichtenthal et al. [43], USA	CARS: overall fear, problems (health worries, womanhood worries, role worries, and death worries)	Outcome	Pretest (T0), posttest (T1), 3-month f/u (T2)	Among the subscales, the CARS-Health worries showed a significant time x condition interaction, and there was reliable improvement in health worries from the baseline to the follow-up for the intervention (AIM-FBCR) group.
Tomei et al. [44], Canada	FCRI: total	Outcome	Baseline (T0): control group only, pretest (T1), posttest (T2), 3-month f/u (T3)	There was a significant interaction effect on FCR: the CBT group showed greater reductions in FCR than the control group, and most changes were maintained at the 3-month follow-up.
van Helmondt et al. [45], The Netherlands	FCRI: severity, psychological distress, coping strategies, & functioning impairments	Outcome	Baseline (T0), posttest (T1), 9-month f/u (T2)	There was no effect of CBT-based online self-help training in reducing FCR in breast cancer survivors compared with that of CAU at posttest and 9-month follow-up.
Heinrichs et al. [46], Germany	FoP-Q-SF	Outcome	Pretest (T0), posttest (T1), 6-month f/u (T2), 12-month f/u (T3)	Patients in the CBT intervention group showed a significantly greater decline in FCR from pre- to post assessment (time x group x sex) than the control group. During long-term follow-up, patients in the control group showed a significant linear decline, while their CBT group counterparts maintained their gains.
Shields et al. [47], USA	CARS: problems (health, womanhood, role, death, and parenting)	Outcome	Baseline (T0), posttest (T1), 1-week f/u (T2), 2-month f/u (T3)	The intervention group showed greater reductions in FCR scores than the control group over time, but the group differences were not statistically significant.
Germino et al. [48], USA	CARS: overall	Outcome	Baseline (T0), 4~6 months postbaseline (T1), 8~10 months postbaseline (T2)	The intervention group had a larger decrease in FCR than the control group, but the result was not statistically significant.
Merckaert et al. [49], Belgium	FCRI: triggers, severity, psychological distress, coping strategies, functioning impairments, insight, and reassurance	Outcome	Pretest (T0), posttest (T1)	Compared with patients in the control group, patients in the CBT group reported greater use of FCR-related coping strategies and greater reduction in FCR-related psychological distress.

### Quality appraisal

Table 5 and Additional file 1 summarize the results of the study quality assessment. The quality of reporting of RCTs of the included studies was assessed using the CONSORT 2010 checklist guidelines. Four items of the checklist were excluded from the analyses because they were not applicable. Not all of the studies complied with the CONSORT 2010 checklist. The average percentage of articles that reported each applicable item on the checklist was 77.3.

**Table 5:** Average reporting percentages for the CONSORT 2010 checklist items

Section/Topic	Item No	Checklist Item	Yes N (%)
**Title and abstract**			
	1a	Identification as a randomized trial in the title	14(82.4)
	1b	Structured summary of trial design, methods, results, and conclusions	17(100.0)
**Introduction**			
Background and objectives	2a	Scientific background and explanation of rationale	17(100.0)
	2b	Specific objectives or hypotheses	17(100.0)
**Methods**			
Trial design	3a	Description of trial design (such as parallel, factorial) including allocation ratio	12(70.6)
	3b	Important changes to methods after trial commencement (such as eligibility criteria), with reasons	1(5.9)
Participants	4a	Eligibility criteria for participants	16(94.1)
	4b	Settings and locations where the data were collected	12(70.6)
Interventions	5	The interventions for each group with sufficient details to allow replication, including how and when they were actually administered	17(100.0)
Outcomes	6a	Completely defined prespecified primary and secondary outcome measures, including how and when they were assessed	17(100.0)
	6b	Any changes to trial outcomes after the trial commenced, with reasons	N.A.
Sample size	7a	How sample size was determined	11(64.7)
	7b	When applicable, explanation of any interim analyses and stopping guidelines	1(5.9)
Randomization:			
Sequence generation	8a	Method used to generate the random allocation sequence	16(94.1)
	8b	Type of randomization; details of any restriction (such as blocking and block size)	12(70.6)
Allocation concealment mechanism	9	Mechanism used to implement the random allocation sequence (such as sequentially numbered containers), describing any steps taken to conceal the sequence until interventions were assigned	6(35.3)
Implementation	10	Who generated the random allocation sequence, who enrolled participants, and who assigned participants to interventions	10(58.8)
Blinding	11a	If done, who was blinded after assignment to interventions (for example, participants, care providers, those assessing outcomes) and how	10(58.8)
	11b	If relevant, description of the similarity of interventions	N.A.
Statistical methods	12a	Statistical methods used to compare groups for primary and secondary outcomes	17(100.0)
	12b	Methods for additional analyses, such as subgroup analyses and adjusted analyses	9(52.9)
**Results**			
Participant flow (a diagram is strongly recommended)	13a	For each group, the numbers of participants who were randomly assigned, received intended treatment, and were analyzed for the primary outcome	17(100.0)
	13b	For each group, losses and exclusions after randomization, together with reasons	13(76.5)
Recruitment	14a	Dates defining the periods of recruitment and follow-up	12(70.6)
	14b	Why the trial ended or was stopped	N.A.
Baseline data	15	A table showing baseline demographic and clinical characteristics for each group	14(82.4)
Numbers analyzed	16	For each group, number of participants (denominator) included in each analysis and whether the analysis was by original assigned groups	17(100.0)
Outcomes and estimation	17a	For each primary and secondary outcome, results for each group, and the estimated effect size and its precision (such as 95% confidence interval)	13(76.5)
	17b	For binary outcomes, presentation of both absolute and relative effect sizes is recommended	N.A.
Ancillary analyses	18	Results of any other analyses performed, including subgroup analyses and adjusted analyses, distinguishing prespecified from exploratory	9(52.9)
Harms	19	All important harms or unintended effects in each group	0(0.0)
**Discussion**			
Limitations	20	Trial limitations, addressing sources of potential bias, imprecision, and, if relevant, multiplicity of analyses	17(100.0)
Generalizability	21	Generalizability (external validity, applicability) of the trial findings	2(11.8)
Interpretation	22	Interpretation consistent with results, balancing benefits and harms, and considering other relevant evidence	17(100.0)
**Other information**			
Registration	23	Registration number and name of trial registry	12(70.6)
Protocol	24	Where the full trial protocol can be accessed, if available	7(41.2)
Funding	25	Sources of funding and other support (such as supply of drugs), role of funders	15(88.2)
**Average percentage**			**77.3**

Among the six sections of the checklist, the included RCT studies had the highest average reporting percentage for the items related to the introduction (100%), followed by those related to the title and abstract (91.2%), discussion (70.6%), the results (69.9%), other information (66.7%) and methods (65.6%). Ten of the 33 applicable items on the checklist were reported by 100% of the included studies, while ten items were reported by less than 60% of the RCT studies. Specifically, only a few studies reported the items on the important changes to methods after trial commencement with reasons (5.9%), the explanation of any interim analyses and stopping guidelines (5.9%), or generalizability (11.8%). None of the studies reported important harms or unintended effects (0%).

In general, the studies of face-to-face CBTs with an intervention duration of at least 1 month were more likely to provide detailed descriptions according to the checklist and to comply with the CONSORT 2010 guidelines. In contrast, the studies of telephone-based CBTs with a single session or a few sessions did not provide sufficient information regarding the CONSORT 2010 items, and they especially underreported issues such as trial design, the allocation concealment mechanism, blinding, recruitment, registration, and protocol.

## Discussion

FCR is very common among BCSs and can lead to other adverse psychosocial outcomes including unmet needs, depression, maladaptive coping behaviors, and low quality of life [8, 12, 13], in the recovery process of cancer. The primary objectives of this study were to systematically review studies of CBT interventions to alleviate FCR among BCSs and evaluate these interventions focusing on the content and methodological aspects of the interventions, the FCR-related outcomes, and the level of adherence to the CONSORT 2010 guidelines.

The current study revealed that the included interventions were comparable to each other in terms of the study design and methodology of CBTs (e.g., RCTs, selection bias, external validity), but these interventions differed considerably in overall intervention structure (e.g., length and intensity). First, approximately two-thirds of CBTs adopted a group format with four to eight sessions, and group treatment formats were shown to have better outcomes in reducing FCR scores than individual formats. These results are inconsistent with previous studies. Tatrow and Montgomery [55] reported in a meta-analysis study that individual CBT approaches showed larger effects on distress and pain in BCSs than group interventions. Other studies have reported that CBT in both group and individual formats is an effective intervention for women with breast cancer [56, 57]. Considering social support among group members and the cost-effectiveness of having a larger number of study participants, group-based interventions may have more benefits than individual therapies [58]. However, this systematic review examined a small number of studies, so the findings should be interpreted with caution. Future research including large samples comparing individual and group CBT formats is warranted to further investigate this issue.

Second, most studies used face-to-face delivery methods, and only a few studies employed either telephone- or internet-based interventions. Some previous studies showed that online-based interventions can be as effective as face-to-face treatments [59], but others claimed that supplementary approaches (e.g., professional support via face-to-face or online, standard telephone or email reminders) are necessary to compensate for the limitations of online-based interventions [60, 61]. Thus, the question of which method is more effective in reducing FCR symptoms among BCSs remains. Considering the ongoing COVID-19 pandemic, it is recommended to expand the scope of delivery methods and incorporate web- and/or mobile-based interventions into the traditional face-to-face method in the future. In addition, although all the included studies minimized selection biases by utilizing RCTs, most studies showed a lack of generalizability. For instance, the majority of studies recruited homogeneous samples (e.g., non-Hispanic Whites recruited from 1 to 2 medical centers), which limited the generalizability of the study findings to other ethnic groups in other countries.

Third, the included studies used various FCR measures and reported promising results in reducing FCR scores. Most studies utilized either the CARS or FCRI, while one study used both the CWS and FCRI [24]. Many studies chose different sets of CARS or FCRI subscales, making it difficult to compare them to one another. In this systematic review, the FCRI was the most frequently used measure, but no gold standard measurements of FCR have been reported in the literature [6]. In terms of FCR outcomes, the study findings showed the effectiveness of CBTs on FCR for BCSs and suggested which approaches hold promise for reducing FCR. Specifically, face-to-face group sessions with at least a one-month intervention duration were more effective in reducing FCR scores than those with brief online or telephone delivery methods, which is consistent with the findings of a prior systematic review [34]. Prior mental health research [59] has documented that web-based interventions have equivalent effects to their face-to-face counterparts, but there is not enough evidence for FCR. Tauber et al. [34] suggested that individually tailored psychological interventions with different treatment components would be beneficial for reducing FCR.

Fourth, the average reporting percentage for the items of the CONSORT 2010 checklist was 77.3, and face-to-face CBTs with an intervention duration of at least 1 month were more likely to comply with the CONSORT 2010 guidelines. Although the included studies generally complied with most of the reporting criteria, the reporting for items in the methods and other information sections needed much improvement, which was an issue mentioned in the previous literature [62]. In particular, studies of very brief CBTs delivered via telephone insufficiently reported information for the CONSORT 2010 items, including trial design, allocation concealment mechanism, blinding, registration, and protocol. A lack of this essential information may be due to limited available space in the manuscript (i.e., word count limit) or a lack of time. The process of randomization itself can pose a challenge to interventionists, and the importance of including and reporting all the detailed information may not be fully appreciated [62]. However, it should be acknowledged that compliance with the relevant reporting guidelines contributes to improving the overall quality of manuscripts and disseminating rigorous and reliable outcomes.

### Implications for future practice and research

The findings of the current study have important clinical and research applications in several ways. First, by focusing on BCSs, the current study reduces heterogeneity issues caused by the inclusion of all cancer types. It is critical to understand FCR-related experiences among BCSs because they often report problems caused by cancer during the treatment process, such as high levels of uncertainty, fear, and concerns related to womanhood, body image, or relationships [63]. Knowledge of the possible detrimental effects of high levels of FCR will be helpful for clinicians to broaden their perspectives and be prepared to provide adequate support for BCSs. Second, this comprehensive systematic review provides evidence-based information on the differences among various types of CBT interventions and the quality of reporting on RCTs. Based on this information, clinicians can determine which approach is most effective in minimizing the negative effects of FCR and how to design interventions for BCSs. Finally, it is noticeable that an active control group such as supportive-experiential group therapy [23] outshined a control group, although its effectiveness was not as high as that of the CBT intervention. This implies the potential for considering such a comparison group as an alternative treatment condition in future research.

### Study limitations

There are several limitations to this review. First, the current study included 17 RCT studies that recruited relatively homogenous samples (middle-aged non-Hispanic White women who more recently completed treatment), which limited the generalizability of the findings. CBT interventionists must try to recruit diverse populations, including different age groups and individuals with diverse ethnic/racial backgrounds. Second, the authors excluded CBTs with quasi-experimental or qualitative research designs from this systematic review; therefore, the findings may provide limited information in this regard. It would be informative to include such studies and compare their findings with those of well-designed RCTs in future studies. Finally, two RCT studies recruited cancer survivors in stages 1 to 4, but no specific information was available on how different cancer stages were associated with FCR-related outcomes. It is possible that cancer survivors in stage 4 are more likely to confuse fear of disease progression for FCR than those in other stages. Thus, these findings should be taken with caution.

## Conclusion

This comprehensive systematic review evaluated different types of CBT interventions on FCR outcomes as well as the quality of reporting of RCTs using the PRISMA guidelines and the CONSORT 2010 checklist. Seventeen CBT studies were included, and the study results revealed that face-to-face RCTs with at least one month of intervention duration showed better FCR outcomes and higher quality of reporting than RCTs with brief online or telephone delivery methods. Future FCR-related studies should include a broader population from multiple centers to ensure generalizability and adhere to the reporting guidelines in the preparation of manuscripts.

## Supplementary Information

**Additional file 1.**

## Data Availability

Not applicable.

## Abbreviations

FCR: Fear of cancer recurrence
BCSs: Breast cancer survivors
RCTs: Randomized controlled trials
CBTs: Cognitive behavioral therapies
PRISMA: Preferred reporting items for systematic reviews and meta-analyses
CONSORT: Consolidated standards of reporting trials
CWS: Cancer worry scale
CARS: Concerns about recurrence scale
FCRI: Fear of cancer recurrence inventory
FoP-Q-SF: Fear of progression questionnaire
QLACS: Quality of life in adult cancer survivors
