# Supplementary material for: Cognitive behavioral therapy for reducing fear of cancer recurrence (FCR) among breast cancer survivors: a systematic review of the literature
Source: BMC Cancer. 2022 Feb 28;22:217. doi: 10.1186/s12885-021-08909-y (PMC8883021; doi:10.1186/s12885-021-08909-y)
Supplement: Supplementary file 1 — Additional file 1. [file 12885_2021_8909_MOESM1_ESM.docx]

**Appendix 1** Study quality assessment based on CONSORT 2010 checklist

| Section/Topic | Item No | Yes | No |
| --- | --- | --- | --- |
| **Title and abstract** | |  |  |
|  | 1a | Bower et al. [36], Butow et al. [26], Dodds et al. [38], Gonzalez-Hernandez et al. [39], Heinrichs et al. [45], Johns et al. [37], Lengacher et al. [27, 40], Lichtenthal et al. [42], Merckaert et al. [48], Park et al. [41], Tomei et al. [43], van de Wal et al. [24], van Helmondt et al. [44] | Germino et al. [47], Herschbach et al. [23], Shields et al. [46] |
|  | 1b | Bower et al. [36], Butow et al. [26], Dodds et al. [38], Germino et al. [47], Gonzalez-Hernandez et al. [39], Heinrichs et al. [45], Herschbach et al. [23], Johns et al. [37], Lengacher et al. [27, 40], Lichtenthal et al. [42], Merckaert et al. [48], Park et al. [41], Shields et al. [46], Tomei et al. [43], van de Wal et al. [24], van Helmondt et al. [44] | None |
| **Introduction** |  |  |  |
| Background and objectives | 2a | Bower et al. [36], Butow et al. [26], Dodds et al. [38], Germino et al. [47], Gonzalez-Hernandez et al. [39], Heinrichs et al. [45], Herschbach et al. [23], Johns et al. [37], Lengacher et al. [27, 40], Lichtenthal et al. [42], Merckaert et al. [48], Park et al. [41], Shields et al. [46], Tomei et al. [43], van de Wal et al. [24], van Helmondt et al. [44] | None |
|  | 2b | Bower et al. [36], Butow et al. [26], Dodds et al. [38], Germino et al. [47], Gonzalez-Hernandez et al. [39], Heinrichs et al. [45], Herschbach et al. [23], Johns et al. [37], Lengacher et al. [27, 40], Lichtenthal et al. [42], Merckaert et al. [48], Park et al. [41], Shields et al. [46], Tomei et al. [43], van de Wal et al. [24], van Helmondt et al. [44] | None |
| **Methods** |  |  |  |
| Trial design | 3a | Bower et al. [36], Butow et al. [26], Dodds et al. [38], Heinrichs et al. [45], Lengacher et al. [27, 40], Lichtenthal et al. [42], Merckaert et al. [48], Park et al. [41], Tomei et al. [43], van de Wal et al. [24], van Helmondt et al. [44] | Germino et al. [44], Gonzalez-Hernandez et al. [49], Herschbach et al. [23], Johns et al. [47], Shields et al. [40] |
|  | 3b | Lichtenthal et al. [42], | Bower et al. [36], Butow et al. [26], Dodds et al. [38], Germino et al. [47], Gonzalez-Hernandez et al. [39], Heinrichs et al. [45], Herschbach et al. [23], Johns et al. [37], Lengacher et al. [27, 40], Merckaert et al. [48], Park et al. [41], Shields et al. [46], Tomei et al. [43], van de Wal et al. [24], van Helmondt et al. [44] |
| Participant | 4a | Bower et al. [36], Butow et al. [26], Dodds et al. [38], Gonzalez-Hernandez et al. [39], Heinrichs et al. [45], Herschbach et al. [23], Johns et al. [37], Lengacher et al. [27, 40], Lichtenthal et al. [42], Merckaert et al. [48], Park et al. [41], Shields et al. [46], Tomei et al. [43], van de Wal et al. [24], van Helmondt et al. [44] | Germino et al. [47] |
|  | 4b | Bower et al. [36], Butow et al. [26], Dodds et al. [38], Gonzalez-Hernandez et al. [39], Heinrichs et al. [45], Johns et al. [37], Lengacher et al. [27, 40], Park et al. [41], Tomei et al. [43], van de Wal et al. [24], van Helmondt et al. [44] | Germino et al. [47], Herschbach et al. [23], Lichtenthal et al. [42], Merckaert et al. [48], Shields et al. [40] |
| Interventions | 5 | Bower et al. [36], Butow et al. [26], Dodds et al. [38], Germino et al. [47], Gonzalez-Hernandez et al. [39], Heinrichs et al. [45], Herschbach et al. [23], Johns et al. [37], Lengacher et al. [27, 40], Lichtenthal et al. [42], Merckaert et al. [48], Park et al. [41], Shields et al. [46], Tomei et al. [43], van de Wal et al. [24], van Helmondt et al. [44] | None |

(Continues)

| Section/Topic | Item No | Yes | No |
| --- | --- | --- | --- |
| Outcomes | 6a | Bower et al. [36], Butow et al. [26], Dodds et al. [38], Germino et al. [47], Gonzalez-Hernandez et al. [39], Heinrichs et al. [45], Herschbach et al. [23], Johns et al. [37], Lengacher et al. [27, 40], Lichtenthal et al. [42], Merckaert et al. [48], Park et al. [41], Shields et al. [46], Tomei et al. [43], van de Wal et al. [24], van Helmondt et al. [44] | None |
|  | 6b | N.A. | N.A. |
| Sample size | 7a | Bower et al. [36], Butow et al. [26], Dodds et al. [38], Gonzalez-Hernandez et al. [39], Herschbach et al. [23], Lengacher et al. [27], Lichtenthal et al. [42], Merckaert et al. [48], Park et al. [41], Tomei et al. [43], van de Wal et al. [24] | Germino et al. [47], Heinrichs et al. [45], Johns et al. [37], Lengacher et al. [40], Shields et al. [46], van Helmondt et al. [44] |
|  | 7b | Merckaert et al. [48] | Bower et al. [36], Butow et al. [26], Dodds et al. [38], Germino et al. [47], Gonzalez-Hernandez et al. [39], Heinrichs et al. [45], Herschbach et al. [23], Johns et al. [37], Lengacher et al. [27, 40], Lichtenthal et al. [42], Park et al. [41], Shields et al. [46], Tomei et al. [43], van de Wal et al. [24], van Helmondt et al. [44] |
| Randomization: |  |  |  |
| Sequence generation | 8a | Bower et al. [36], Butow et al. [26], Dodds et al. [38], Germino et al. [47], Gonzalez-Hernandez et al. [39], Heinrichs et al. [45], Herschbach et al. [23], Johns et al. [37], Lengacher et al. [27, 40], Lichtenthal et al. [42], Merckaert et al. [48], Park et al. [41], Tomei et al. [43], van de Wal et al. [24], van Helmondt et al. [44] | Shields et al. [46] |
|  | 8b | Bower et al. [36], Butow et al. [26], Dodds et al. [38], Germino et al. [47], Heinrichs et al. [45], Johns et al. [37], Lengacher et al. [27, 40], Lichtenthal et al. [42], Park et al. [41], van de Wal et al. [24], van Helmondt et al. [44] | Gonzalez-Hernandez et al. [47], Herschbach et al. [23], Merckaert et al. [48], Shields et al. [46], Tomei et al. [43] |
| Allocation concealment mechanism | 9 | Bower et al. [36], Heinrichs et al. [45], Johns et al. [37], Merckaert et al. [48], Tomei et al. [43], van Helmondt et al. [44] | Butow et al. [26], Dodds et al. [38], Germino et al. [47], Gonzalez-Hernandez et al. [39], Herschbach et al. [23], Lengacher et al. [27, 40], Lichtenthal et al. [42], Park et al. [41], Shields et al. [46], van de Wal et al. [24] |
| Implementation | 10 | Butow et al. [26], Dodds et al. [38], Germino et al. [47], Heinrichs et al. [45], Johns et al. [37], Park et al. [41], Shields et al. [46], Tomei et al. [43], van de Wal et al. [24], van Helmondt et al. [44] | Bower et al. [36], Gonzalez-Hernandez et al. [47], Herschbach et al. [23], Lengacher et al. [27, 40], Lichtenthal et al. [42], Merckaert et al. [48] |
| Blinding | 11a | Butow et al. [26], Dodds et al. [38], Gonzalez-Hernandez et al. [47], Heinrichs et al. [45], Johns et al. [37], Lengacher et al. [40], Lichtenthal et al. [42], Tomei et al. [43], van de Wal et al. [24], van Helmondt et al. [44] | Bower et al. [36], Germino et al. [47], Herschbach et al. [23], Lengacher et al. [27], Merckaert et al. [48], Park et al. [41], Shields et al. [46] |
|  | 11b | N.A. | N.A. |
| Statistical methods | 12a | Bower et al. [36], Butow et al. [26], Dodds et al. [38], Germino et al. [47], Gonzalez-Hernandez et al. [39], Heinrichs et al. [45], Herschbach et al. [23], Johns et al. [37], Lengacher et al. [27, 40], Lichtenthal et al. [42], Merckaert et al. [48], Park et al. [41], Shields et al. [46], Tomei et al. [43], van de Wal et al. [24], van Helmondt et al. [44] | None |
|  | 12b | Butow et al. [26], Dodds et al. [38], Germino et al. [47], Heinrichs et al. [45], Lengacher et al. [27, 40] Shields et al. [46], van de Wal et al. [24], van Helmondt et al. [44] | Bower et al. [36], Gonzalez-Hernandez et al. [47], Herschbach et al. [23], Johns et al. [37], Lichtenthal et al. [42], Merckaert et al. [48], Park et al. [41], Tomei et al. [43] |

**Appendix 1** (Continued)

(Continues)

**Appendix 1** (Continued)

| Section/Topic | Item No | Yes | No |
| --- | --- | --- | --- |
| **Results** |  |  |  |
| Participant flow (a diagram is strongly recommended) | 13a | Bower et al. [36], Butow et al. [26], Dodds et al. [38], Germino et al. [47], Gonzalez-Hernandez et al. [39], Heinrichs et al. [45], Herschbach et al. [23], Johns et al. [37], Lengacher et al. [27, 40], Lichtenthal et al. [42], Merckaert et al. [48], Park et al. [41], Shields et al. [46], Tomei et al. [43], van de Wal et al. [24], van Helmondt et al. [44] | None |
|  | 13b | Bower et al. [36], Butow et al. [26], Dodds et al. [38], Gonzalez-Hernandez et al. [39], Heinrichs et al. [45], Johns et al. [37], Lengacher et al. [27], Lichtenthal et al. [42], Merckaert et al. [48], Park et al. [41], Shields et al. [46], Tomei et al. [43], van de Wal et al. [24] | Germino et al. [47], Herschbach et al. [23], Lengacher et al. [40], van Helmondt et al. [44] |
| Recruitment | 14a | Bower et al. [36], Butow et al. [26], Dodds et al. [38], Gonzalez-Hernandez et al. [39], Heinrichs et al. [45], Lengacher et al. [27, 40], Lichtenthal et al. [42], Merckaert et al. [48], Park et al. [41], van de Wal et al. [24], van Helmondt et al. [44] | Germino et al. [47], Herschbach et al. [23], Johns et al. [37], Shields et al. [46], Tomei et al. [43], |
|  | 14b | N.A. | N.A. |
| Baseline data | 15 | Bower et al. [36], Butow et al. [26], Dodds et al. [38], Gonzalez-Hernandez et al. [39], Heinrichs et al. [45], Herschbach et al. [23], Johns et al. [37], Lengacher et al. [27], Lichtenthal et al. [42], Merckaert et al. [48], Park et al. [41], Shields et al. [46], Tomei et al. [43], van Helmondt et al. [44] | Germino et al. [47], Lengacher et al. [40], van de Wal et al. [24] |
| Numbers analyzed | 16 | Bower et al. [36], Butow et al. [26], Dodds et al. [38], Germino et al. [47], Gonzalez-Hernandez et al. [39], Heinrichs et al. [45], Herschbach et al. [23], Johns et al. [37], Lengacher et al. [27, 40], Lichtenthal et al. [42], Merckaert et al. [48], Park et al. [41], Shields et al. [46], Tomei et al. [43], van de Wal et al. [24], van Helmondt et al. [44] | None |
| Outcomes and estimation | 17a | Butow et al. [26], Dodds et al. [38], Gonzalez-Hernandez et al. [39], Herschbach et al. [23], Johns et al. [37], Lengacher et al. [27], Lichtenthal et al. [42], Merckaert et al. [48], Park et al. [41], Shields et al. [46], Tomei et al. [43], van de Wal et al. [24], van Helmondt et al. [44] | <Studies that did not report any effect sizes and 95% CI>  Bower et al. [36], Germino et al. [47], Heinrichs et al. [45], Lengacher et al. [40] |
|  | 17b | N.A. | N.A. |
| Ancillary analyses | 18 | Butow et al. [26], Dodds et al. [38], Germino et al. [47], Heinrichs et al. [45], Lengacher et al. [27, 40], Shields et al. [46], van de Wal et al. [24], van Helmondt et al. [44] | Bower et al. [36], Gonzalez-Hernandez et al. [39], Herschbach et al. [23], Johns et al. [37], Lichtenthal et al. [42], Merckaert et al. [48], Park et al. [41], Tomei et al. [43] |
| Harms | 19 | None | Bower et al. [36], Butow et al. [26], Dodds et al. [38], Germino et al. [47], Gonzalez-Hernandez et al. [39], Heinrichs et al. [45], Herschbach et al. [23], Johns et al. [37], Lengacher et al. [27, 40], Lichtenthal et al. [42], Merckaert et al. [48], Park et al. [41], Shields et al. [46], Tomei et al. [43], van de Wal et al. [24], van Helmondt et al. [44] |
| **Discussion** |  |  |  |
| Limitations | 20 | Bower et al. [36], Butow et al. [26], Dodds et al. [38], Germino et al. [47], Gonzalez-Hernandez et al. [39], Heinrichs et al. [45], Herschbach et al. [23], Johns et al. [37], Lengacher et al. [27, 40], Lichtenthal et al. [42], Merckaert et al. [48], Park et al. [41], Shields et al. [46], Tomei et al. [43], van de Wal et al. [24], van Helmondt et al. [44] | None |
| Generalizability | 21 | Butow et al. [26], Germino et al. [47] | Bower et al. [36], Dodds et al. [38], Gonzalez-Hernandez et al. [39], Heinrichs et al. [45], Herschbach et al. [23], Johns et al. [37], Lengacher et al. [27, 40], Lichtenthal et al. [42], Merckaert et al. [48], Park et al. [41], Shields et al. [46], Tomei et al. [43], van de Wal et al. [24], van Helmondt et al. [44] |

(Continues)

**Appendix 1** (Continued)

| Section/Topic | Item No | Yes | No |
| --- | --- | --- | --- |
| Interpretation | 22 | Bower et al. [36], Butow et al. [26], Dodds et al. [38], Germino et al. [47], Gonzalez-Hernandez et al. [39], Heinrichs et al. [45], Herschbach et al. [23], Johns et al. [37], Lengacher et al. [27, 40], Lichtenthal et al. [42], Merckaert et al. [48], Park et al. [41], Shields et al. [46], Tomei et al. [43], van de Wal et al. [24], van Helmondt et al. [44] | None |
| **Other information** |  |  |  |
| Registration | 23 | Bower et al. [36], Butow et al. [26], Gonzalez-Hernandez et al. [39], Heinrichs et al. [45], Johns et al. [37], Lengacher et al. [27], Lichtenthal et al. [42], Merckaert et al. [48], Park et al. [41], Tomei et al. [43], van de Wal et al. [24], van Helmondt et al. [44] | Dodds et al. [38], Germino et al. [47], Herschbach et al. [23], Lengacher et al. [40], Shields et al. [46] |
| Protocol | 24 | Bower et al. [36], Butow et al. [26], Dodds et al. [38], Gonzalez-Hernandez et al. [39], Heinrichs et al. [45], van de Wal et al. [24], van Helmondt et al. [44] | Germino et al. [47], Herschbach et al. [23], Johns et al. [37], Lengacher et al. [27, 40], Lichtenthal et al. [42], Merckaert et al. [48], Park et al. [41], Shields et al. [46], Tomei et al. [43] |
| Funding | 25 | Bower et al. [36], Butow et al. [26], Gonzalez-Hernandez et al. [39], Heinrichs et al. [45], Herschbach et al. [23], Johns et al. [37], Lengacher et al. [27, 40], Lichtenthal et al. [42], Merckaert et al. [48], Park et al. [41], Shields et al. [46], Tomei et al. [43], van de Wal et al. [24], van Helmondt et al. [44] | Dodds et al. [38], Germino et al. [47] |
